# The Overeducated Woman and the Race Question

**Published:** 1904-10

**Authors:** 


### The Overeducated Woman and the Race Question.

The native-born American woman has been the subject of discussion *ad nauseum* indeed. It seems never, or almost never, to occur to the medical profession that the condition of the male population has anything to do with the past, present, or future of the race; all depends upon the overtaxed brain of our educated women.

Has medical science reached that point where one can hope to see figs grow on thistles? Is there a law in physics that proves that we can hope for perfect development from blighted, contaminated seed; or can we transfer all weakness and inherited taint to the male population, and the female has no share but to rise out of the mire a glorified intellectual being with brain power sufficient to control herself and help control the universe.

When statistics tell us that ninety out of every one hundred men are or have at some time been afflicted with gonorrhea, the deduction one would naturally draw is: that only 10 per cent of the male population are capable of reproducing a normal healthy being, and yet almost nothing is said of this phase of human development, but all blame placed to the overtaxed brain of intelligent women.

The cry is what can be done for the boys. Teach them that they can be as bright, energetic, and resourceful as their sisters; that they have as much brain power, if properly cultivated and guided, and can attain heights morally, mentally, and physically impossible for those whose vitality is exhausted by wrong living.

From what source, if not from the medical profession shall this change come? Come it must, and I for one shall be glad to see the profession, rather than these overtaxed women, undertake the work.—*Josephine Kingsley, M. D., San Antonio, Texas, in N. Y. Medical Record, August 6, 1904.*

---

A Medical College for Negroes has been opened at Smithville, Texas, a town of 3000 in Bastrop county. In connection with the "Smithville Medical and Surgical Institute," as it is called, there will be a training school for colored nurses. The movement, if honestly and efficiently executed, is praiseworthy and should be emulated in the larger cities. We clip the following from the lay press:

"The director of the institute is Dr. T. Adolf Jones, a graduate of the College of Physicians and Surgeons, Boston, Mass., lecturer on embryology in the Flint Medical College of New Orleans University and at one time acting resident surgeon of the Sarah Goodridge Hospital and Nurse Training School of New Orleans University. Mrs. Jones, who was at one time lecturer on household economics in the same institution, will be in charge of the resident nurses. Any reputable surgeon having colored patients will be allowed to operate in the building, and such patients will be nursed by the staff. Patients who desire will be attended to by the resident surgeon.

"Free lectures on preventive medicine will be delivered at stated periods, and free treatment will be given the deserving poor on Saturdays.

"The nurse training department includes instruction in midwifery, and students who so desire will be specially prepared for the State Board.

"Dr. Jones is of the opinion that the high mortality of consumption and syphilis in the negro race can be greatly diminished by instructing the colored people through specially prepared colored nurses, male or females, and to that end will make free house-to-house nursing a part of the curriculum of the students of the school."

